# Gender motivational gap and contribution of different teaching approaches to female students’ motivation to learn physics

**DOI:** 10.1038/s41598-022-23151-7

**Published:** 2022-10-29

**Authors:** Branka Radulović, Vera Županec, Maja Stojanović, Spomenka Budić

**Affiliations:** 1 Department of Physics, University of Novi Sad, Faculty of Sciences, Novi Sad, Republic of Serbia; 2 Department of Biology and Ecology, University of Novi Sad, Faculty of Sciences, Novi Sad, Republic of Serbia; 3 College of Vocational Studies for the Education of Preschool Teachers and Sports Trainers, Subotica, Republic of Serbia; 4 Educons University in Sremska Kamenica, Sremska Kamenica, Republic of Serbia

**Keywords:** Physics, Techniques and instrumentation, Psychology and behaviour

## Abstract

This research focuses on potential gender differences in motivation to learn Physics with the aim to determine the weakest female motivational components to learn Physics and the contribution of different teaching approaches (using real and virtual experiments) on those components and motivation for learning Physics in general. These two approaches were chosen as the most commonly used approaches in physics but without clear compared indication on females’ motivation. The standardized questionnaire SMTSL (Student’s Motivation towards Science Learning) is used for the measurements. The results show that for female students the weakest motivational components to learn Physics are the importance of Physics as a science and self-efficacy. Virtual experiments contribute more to females’ motivation to learn Physics than applying real experiments. The female students who used real experiments show fear of being laughed at by their male peers and express doubt in their self-knowledge. Although the applied approaches cause some improvements in female students’ self-efficacy, they are not statistically significant. Research results suggest that teachers need to apply such teaching approaches that engage girls and encourage their learning and development in order to improve their self-efficacy and other motivational components.

## Introduction

Feminist strategies for diversifying STEM have brought awareness to sex-segregation, but they have not changed the fields themselves^1^. Therefore, in numerous research and longitudinal studies^2^, the problem of women’s participation in Science, especially in Physics, is still recognized. According to NCSES^2^, “Physics has the lowest share of women degree recipients within the broad field of physicsal sciences” and at all degree levels, despite their (females’) qualifications. The reason for such a small presence of women in Physics can be found in their interest in Physics in primary and secondary education. Numerous studies have shown that gender is one of the largest determinants of Physics uptake at A level^3,4^. According to these reports, boys are more interested in Physics than girls. The most common reasons are related to differences in the way of thinking or learning, competence beliefs, the absence of role models, the sense of belonging, perception of physical sciences, classroom experience and learning styles^5,6^. Classroom processes, such as social context and classroom experience, are important predictors of students’ learning^7^. Therefore it appears necessary to find teaching approaches that will encourage students’, expecial females’ interest in learning Physics and their self-confidence, and connect physical laws with examples which are more understandable to female students. Encouraging students’ interest in Physics, and Science in general, will have a positive effect on students’ attitudes towards the subject and their efforts to learn the content^8^. If students develop positive attitudes towards a subject, they will invest more time in researching phenomena and searching for new and specific information, which will eventually result in greater motivation for the subject and the scientific field. However, if students develop negative attitudes or prejudices about a subject, they will hardly engage in it. It therefore implies that students’ dissatisfaction from their previous experiences could predict their interest and affect their choice and decision in science^9,10^. Thus, it can be concluded that the right choice of teaching approaches should increase students’ (in this case, females’) motivation for learning. Accordingly, the current research is aimed at determining the contribution of different teaching approaches (real or virtual experiments) to females’ motivation to learn Physics.

## Theoretical review

The issue of student motivation includes interest, commitment, and identification with a given subject^11^. According to Archer et al.^12^, girls during adolescence lose interest in science because they perceived it as not relevant. Carlone^13^ concludes that adolescent females may try to avoid science activities because they do not identify with it. They are more identificated with people-oriented subjects such as biology while physics is described as unattractive^14^. Therefore, gender identity is recognized as a core element in the development of a sense of self^13^ and one of the most pervasive and enduring influences on personal goals, aspirations, and behavior^13^. The construction of identity is influenced by external factors such as the teacher^6,9^, parents, peers and the social environment^15,16^ that can contribute to the creation or maintenance of a stereotype, such as a stereotype that physics is not for girls. According to Steele and Aronson^17^, a stereotype threat refers to individual fears of confirming, through individual behaviors, a stereotype associated with the group. Therefore, if the student is presented with such teaching approaches that can encourage discrimination of any kind, he/she is likely to adopt them and, based on such behaviours, build beliefs about what he/she is capable or uncapable for.

It is known that women and girls are faced with negative stereotypes about their participation in physical sciences^15–20^, but little is known about how their more significant presence in these sciences can be encouraged. Bearing this in mind, this research intends to determine the influence of two teaching approaches based on experiments which have a central feature in Physics learning on female student motivation to learn Physics. Both approaches (based on virtual and on real experiments) imply students’ active involvement in the learning process through designing and performing experiments^21,22^ and positively influence on students’ knowledge^23,24^; but there is not a comparison of motivational effects which cause these two approaches.

Both approaches are based on collaborative work, which is recognized as support to students’ sense of belonging^12^ and improve students’ performance^23,24^; but collaborative work can also be a reason for increasing stereotypes. According to Delisle et al.^17^, for gender stereotype, three conditions are needed: the existence of a negative stereotype, identifying with the field, and facing with a task within the field related to the stereotype. The existence of stereotypes can be observed in physics textbooks, where examples of women's achievements in physics are rarely given. Also, when illustrating physical phenomena in textbooks, men are often shown performing the given phenomenon^20^. In addition to this slight emphasis on male dominance in physics, within the teaching of physics itself, facing the task is a particularly important and characteristic condition because it can be both computational and experimental. As boys are more skilled in assembling apparatus, especially if it is about electric currents, then approach based on real experiments can encourage doubts among girls about their own skills and knowledge. As students’ motivation to learn a subject is in directly correlates with their identification and self-efficacy^13,25–27^, it appears necessary to determine how these approaches influence females’ motivation to learn Physics. In other words, if students do not see themselves as capable of learning or doing a specific task, they will give it up. According to Bandura, self-efficacy is the assessment of one’s own ability to organize and perform actions necessary to achieve desired goals^26^. Students evaluate their abilities by themselves, how much effort they put into solving the problem, the problem’s difficulty, if they need help or not, etc.^28^. Therefore, they choose to learn something or not.

Due to the complexity of the concept of motivation, in this research, it is viewed through its components: self-efficacy, applying active learning strategies, understanding the importance of Physics as a science, motivation oriented on achievement, and motivation oriented on learning. Thus, a broader picture of the motivational effects of the applied teaching approaches is covered, because, among other things, the ability of female students to perceive physical laws in everyday life is examined. Understanding the importance of some science in everyday life has a special influence on the acceptance and study of this science^26,29^, i.e., if girls perceive that physics contributes to the development of society, i.e. if they perceive the humanistic importance^14^, they will pay more attention to its study. Otherwise, if, through the application of teaching approaches, they are exposed to situations that raise doubts about their own knowledge and skills, they will accept stereotypes and will not make enough effort to master the material, but will immediately characterize it as uninteresting, unimportant and too difficult for them. In order to find pedagogical approaches that will encourage the number of girls in physics, the goal of this research is aimed at examine gender differences in student motivation in order to single out the components that need to be stimulated by teaching approaches (first phase of the research). Then, in the second phase of the research, the contribution of applied teaching approaches to motivation for learning physics in general and individual motivational components will be compared.

## Research methodology

In line with this aim, the following research questions are formulated.

1. What is the lowest female motivational component?
2. How do the laboratory inquiry-based experiments approach (LIBE) and interactive computer-based simulations (ICBS) approaches influence the lowest-rated motivational components and general motivation to learn Physics of female students?

### Sample

The sample of the first phase consisted of a total of 1853 secondary school students from the city of Novi Sad, Serbia. The Raosoft application was used for the calculation of the sample size. As the maximum sample of secondary school students in Serbia was 253,997 students, and our sample of 1853 students represented a convenient sample, since it was higher than 662, which is the threshold defined with the confidence level of 99%. The sample is collected based on a priori power analyses. According to the gender structure, 803 respondents (43.3%) were male students, and 1050 respondents (56.7%) were female students.

The second phase sample consisted of 139 s-grade secondary school female students from Novi Sad, Serbia. These students did not participate in the first phase of the research and were selected by their physics teacher. Female teachers were included in the research. The assumption was that female teachers can serve as role models and have potentially positive influence on female student motivation for learning Physics. We wanted to keep this variable constant because the Nehmeh and Kelly^6^ study showed that teacher influence could encourage or suppress the observed gender gap. As there are more male than female Physics teachers^30^, the aim was to ensure the influence of this variable on the research results, i.e. to eliminate the influence of the teacher gender on the behavior of students in micro groups.

The LIBE group consisted of 70 female students, while the ICBS group consisted of 69 female students. The groups were formed on the basis of already existing classes. The goal of the research and ethical approval were presented to all students, principals and the pedagogical-psychological service. The students volunteered to participate in the research. Students who did not want to participate in the research did everything as students who participated, only their results were not entered into the database.

### Procedure

In order to better understand the problems related to female students’ motivation to learn Physics, the research was divided into two phases. The first phase aims to determine students’ motivation to learn Physics in general and then to observe gender differences in motivational components in order to determine the weakest graded components that need to be influenced upon. This phase was conducted from March to May 2018.

The second phase included a pedagogical experiment with two groups of students. One group used the laboratory inquiry-based experiments (LIBE) approach while the other used the interactive computer-based simulations (ICBS) approach. Each group was further divided into smaller (micro) groups. The micro groups included three students, both male and female. Therefore, the entire classes were divided into micro groups. It was not possible to take care of the gender distribution of each micro group as it depended on the gender distribution of the whole class. Thus, one micro group contained two male and one female student, while in other class micro groups contained two female and one male student. The purpose of creating micro groups was to observe female students’ reactions during the application of the LIBE and ICBS teaching approaches in presence of their male peers. The observation was performed by their teachers as the presence of another observer might have further influenced the students’ behavior. Female teachers presented reports on students’ behavior on a weekly basis (Supplementary information). Students within the micro-group were supposed to discuss how to set up experiments, how to write down the results and calculate the required quantities. In such situations, the students’ behavior was monitored, i.e. whether they chose to record the results and thus acquired a somewhat more passive role, or they wanted to set up experiments and measures of the required size.

All students (male and female) took a motivation test before and after the pedagogical experiment so that all students in the group had a sense of the same level of responsibility and equal engagement. Additionally, a possible exclusion of boys in these motivation tests might have had a negative effect on the quality and accuracy of girls’ responses, as they might have had a feeling of being urged to complete the questionnaire. However, considering the goal of the research, the results of male students were not entered into the database.

The topics chosen for this research are Electrostatics and Direct Current, as they include concepts that can cause a more significant gender difference^31^, and more challenging environment for girls from stereotype side of view, which can reflect on their demotivation for learning Physics. In Serbia, elementary school students are introduced to basic concepts in these areas within a few hours, while in secondary school curriculum these concepts are studied in more detail. In Serbian education system, Physics is an obligatory subject for both elementary and secondary school students. Topic Electrostatics consists of 17 units, while Direct Current consists of 30 units. Within these units, students are trained to solve problems by applying the laws of electrostatics and electrodynamics, calculate the field of charged bodies using Gauss’s theorem, interpret the mechanisms of conducting electricity in metals, electrolytes, and gases, etc. Also, they need to conduct some experiments. In this study, both real and virtual experiments were performed and students demonstrated lines of electric force, Faraday’s cage, or make the electric circuit. All experiments in both groups were the same, but the learning environment differed. The ICBS group used PhET interactive simulations to demonstrate the relationships between phenomena, while the LIBE group used hands-on experiments. Because some research indicates the difference in technology approaches^5^, the ICBS group used already ready-made simulations, which are widely used and recognized, so the results can be compared with other researches. The pedagogical experiment was conducted from January to June 2019.

### Instrument

Due to the complexity of motivation as a theoretical construct^11^, many researchers^32–34^ have tried to find accurate instruments for getting complete information on students’ motivation. Consequently, there are a few instruments for determining students’ motivation. In this research, SMTSL (Student’s Motivation towards Science Learning) questionnaire developed by Tuan et al.^33^ and translated into Serbian language by Olić et al.^29^ has been applied. The questionnaire comprises 29 items subdivided into five subscales: self-efficacy, active learning strategies, understanding the importance of Physics as a science, motivation oriented on achievement, and motivation oriented on learning.

Likert five-point scale was applied for each item so that students could provide their (dis)agreement with the given items. As satisfactory psychometric properties of this questionnaire have been confirmed in numerous papers^29,33,35–37^, it was considered reliable. The Cronbach α coefficient for category self-efficacy was 0.79; the application of active learning strategies was 0.84; appreciation of the importance of Physics was 0.81; motivation oriented to learning was 0.82, and the motivation oriented to the achievement was 0.72, which all confirm validation of the instrument.

Female teachers used an open-ended questionnaire to get information about female students’ reactions during the classes.

### Ethical considerations

The consent of the school principal, Physics teacher, the school board, all participants, and their legal guardians was obtained for the implementation and realization of the research from each school. All students who were a constituent part of the research sample voluntarily accepted their participation in it. They were introduced to the possibility of their own exclusion from the research at any time of its implementation without having any consequences. The study was conducted in accordance with the Declaration of Helsinki, and approved by Director of the Department of Physics, Faculty of Sciences, University of Novi Sad (protocol code 02-6/20; date of approval 18 April 2018).

### Data analysis

Data were analysed through descriptive statistics and advanced statistical models. ANOVA and t-test were used to determine whether there were any statistically significant differences between the means of independent groups. Statistical analysis was performed in SPSS 20.

### Institutional review board statement

The study was conducted in accordance with the Declaration of Helsinki, and approved by Director of the Department of Physics, Faculty of Sciences, University of Novi Sad (protocol code 02-6/20; date of approval 18 April 2018). The students were voluntarily participating in the research, and informed consent to participate in the research was obtained from each participant, their legal guardians, the school principal, Physics teacher, and the school board.

## Research results

ANOVA shows no difference between male and female students in their motivation towards Physics in general, F(d*f* = 1) = 1.026, *p* > 0.05. It was obtained that female (M = 94.08, SD = 14.20) and male students (M = 94.78, SD = 14.91) perceive the same values for their motivation towards Physics. However, there are some differences in observed motivational components (Table 1).

**Table 1:** Gender differences on observed motivational components.

	Active learning strategies		Importance of physics		Motivation oriented to learning		Self-efficacy		Motivation oriented to achievement	
	M	SD	M	SD	M	SD	M	SD	M	SD
Male	29.4	6.1	17.3	4.4	19.7	4.1	16.9	4.1	11.5	3.1
Female	29.4	5.9	16.2	4.4	20.3	4.0	16.0	4.1	12.1	2.8
*t*	− 0.091		5.120**		− 3.095**		4.660**		− 4.820**	
*Eta-square*			0.014		0.005		0.012		0.012	

**p < 0.01.

The obtained results (Table 1) show a small effect of gender differences on the appreciation of the importance of Physics, self-efficacy, motivation oriented to learning and achievement. According to these results, male students were more interested in solving unknown problem situations than females and they had greater confidence in their knowledge. To female students it was very important to get high scores and to have proper knowledge behind the score. Many studies have shown that female students were hardworking and diligent, but they feared certain subjects requiring manual involvement^38^.

According to the results, the appreciation of Physics and self-efficacy should be increased in order to obtain equal motivation of female and male students. With this in mind, the pedagogical experiment was applied to improve these motivational components and raise motivation for learning Physics in general. ANOVA showed no difference between the groups in their motivation towards Physics before the pedagogical experiment, F(d*f* = 1) = 3.392, *p* > 0.05. This means that female students in the LIBE group expressed similar motivation as females in the ICBS group (Table 2). After the pedagogical experiment, ANOVA pointed to the difference between the groups, F(d*f* = 1) = 11.077, *p* < 0.001, eta-square = 0.075. Female students in the LIBE group perceived a lower motivation than the females in the ICBS group. T-test of the paired sample was applied to determine the contribution of the applied teaching approaches to females’ motivation to learn Physics.

**Table 2:** Contribution of applied teaching approaches to females’ motivation to learn Physics.

	Motivation before pedagogical experiment		Motivation after pedagogical experiment		*t*	*Eta-square*
	M	SD	M	SD		
LIBE	87.8	11.8	91.5	11.2	2.699**	0.094
ICBS	83.6	14.9	98.0	11.8	6.220**	0.366

**p < 0.01.

As shown in Table 2, the ICBS approach contributes more significantly to increasing motivation than the LIBE approach. Table 3 shows the exact contribution to the individual motivational components.

**Table 3:** Contribution of applied teaching approaches to observed motivational components.

	LIBE				ICBS			
	M_before_	M_after_	*t*	*Eta-square*	M_before_	M_after_	*t*	*Eta-square*
Active learning strategies	25.3	25.8	1.403	–	20.8	28.7	7.474**	0.451
Importance of Physics	18.3	19.5	2.747**	0.099	14.8	17.8	3.989**	0.190
Motivation oriented to learning	15.3	16.2	9.285**	0.620	10.8	15.0	8.601**	0.510
Self-efficacy	26.5	27.0	0.921	–	22.4	23.0	1.074	–
Motivation oriented to achievement	18.1	15.0	− 3.514**	0.152	19.9	12.2	− 7.041**	0.427

**p < 0.01.

As shown in Table 3, both approaches contributed to the perception of the importance of Physics and students’ higher orientation towards the curriculum, but the differences were more noticeable in the ICBS group. Therefore, female students in the ICBS group seem to connect Physics with everyday problems and analyze them following Physics laws to a greater extent than their LIBE peers. Higher orientation towards understanding the teaching content seems to have caused a decrease in motivation focused only on assessment as a part of external motivation. Also, the ICBS approach has contributed to increasing the use of active learning strategies.

By observing the female students’ behaviour in the classes, females in the LIBE group appeared reluctant to perform the experiments. They preferred to record the measurement results and enumerate the theoretical patterns that supported the performed experiment. During electrostatics experiments, due to the teacher’s support, they had an easier access to the measurement. However, during the experiments with the circuit, they showed greater insecurity and fear of doing something wrong when assembling the circuit and causing a short circuit. Although they had the teacher’s support, they expressed fear that their male peers would laugh at their mistakes.

The female students in the ICBS group had a particular advantage: the possibility of repeating the experiment without damaging the equipment. If they misconnected the electrical circuit and caused a short circuit, they could repeat the same experiment without being noticed by many of their male peers and without causing physicial damage of the devices used in the experiment. This seems to have made the students more relaxed. They did not feel condemnation and possible discrimination from their male peers. The ICBS group of females independently searched the Internet at home, looking for similar experiments, which show their interest in the teaching content.

Nevertheless, neither the ICBS nor the LIBE approach statistically significantly changed the sense of self-efficacy. The feeling of non-identification with Physics is “deeply rooted”, so the applied activities were not sufficient to eliminate persistent self-doubts about their abilities. However, as other motivational components have changed, it is believed that the sense of self-efficacy would change with the support of teachers and after investing more time.

## Discussion

Research has pointed to a problem of the low presence of women in undergraduate Physics courses^6^ which is the reason why their motivation to learn Physics in primary and secondary school should be considered. Some of the reasons are the decline in females’ interest in Physics^39^ which can be influenced by situations or environments which could promote negative stereotypes or females’ non-identification with Physics. In order to determine the contribution of different learning environments on females’ motivation to learn Physics, in present study examined the impact of real and virtual experiments, as the two most common new approaches in teaching Physics. Although laboratory experimental activities have a distinctive and central role in the Physics curriculum and present a unique and powerful opportunity for students to develop their Physics identities by allowing them to experiment and analyze data^40^, they are still not sufficiently applied in Serbia^41^. The reason may refer to the lack of functional Physics laboratories and inappropriate equipment for practical activities. In such a situation, ICBS represents a good solution for overcoming the above problems. This also seems to be one of the reasons for the exponential growth of ICBS in the last few decades. As both approaches are based on collaborative work which could promote doubt in female’s knowledge and skills which are necessary for solving physics problems; the aim of this research was to look at potential gender differences in motivation to learn Physics in order to identify the weakest female motivational components to learn Physics, and to determine the contribution of real and virtual experiments to those components and female students’ motivation to learn Physics in general.

Results of the first phase showed that there were no significant gender differences in student motivation to learn Physics, but it is noticed that male students gave higher scores to the importance of Physics as a science and self-efficacy than females. On the other hand, more female students gave higher scores for motivation oriented to learning and achievement. These results are consistent with the results of Reid and Skryabina^42^. According to them, male students perceived that they liked Science lessons more than females and liked to spend more time on Science because it was interesting to them. Also, the result is in the line with Byman et al.^43^ that Physics as a discipline and school subject is claimed to be highly male-dominated.

In order to reduce the number of external factors that influence the creation or maintenance of stereotypes, that is, they can negatively influence student motivation; teacher gender as a variable was kept as a constant with a potentially positive impact on female motivation to learn Physics. The second phase of the research included classes with female Physics teachers and students divided into two groups, LIBE and ICBS. Each group was further divided into smaller, micro-groups. The micro-groups contained male and female students in order to encourage greater interaction between them during the experiments. Performing experiments, both real and virtual, includes elements of collaborative learning. Greater interaction between them can cause a larger female reaction which indicated the level of female self-confidence. In such situations, the female students’ behavior was monitored by their teacher, i.e. whether they chose to record the results and thus acquired a somewhat more passive role, or they wanted to set up experiments and measures of the required size. In the LIBE group, while the topic Electrostatics was presented, the female students did not show considerable differences in behavior in relation to the boys. They equally participated in measuring physics phenomena, such as demonstrating lines of electric force, Faraday’s cage or conductor capacitance measurement, and recording the results as boys did. However, during the presentation of the topic of Direct current, especially when connecting circuits, their “pull-back” was noticed, i.e. they rather chose to record the results than construct the circuit and measure the physical quantities. No significant changes in girls’ behavior during the pedagogical experiment were observed in the ICBS group. When asked after the class why they had not measured but only recorded the results the LIBE group female students answered that they felt discomfort due to the possibility of being mocked by their male peers. This behaviour pattern can be explained by two factors: unclarity of the learning content and a stronger gender influence triggered by the content unclarity. According to^44^ both high school students and teachers reported that Vectors and Equilibrium of force were easier to grasp than AC Circuits. Similar findings are reported in another study^45^ where preservice physics teachers assessed the topic Mechanics 1 as easiest for understanding, while the topic Direct Current was seen as considerably harder. This was the reason why we decided to choose this topic for our study. If female students’ motivation for learning Physics is increased while acquiring such a demanding topic, then a similar or even more significant effect could be expected in learning other topics that females may seem more familiar and comfortable with, such as the topic of Mechanics, for example, is.

Another explanation for a more passive role of girls in the LIBE group could be a greater pressure upon girls exerted by their male groupmates. The results of Köller et al.^46^ point to a powerful influence of peers, particularly of boys’ influence on girls, as girls tend to show greater fear of being excluded from their group of peers. Similarly, Marchand and Taasoobshirazi^16^ research results suggest that simply being in a typical physics testing situation may be enough to inhibit female performance compared with males. Also, Taasoobshirazi reported that female college students had higher assessment anxiety than their male peers in introductory level Physics^47^. Mujtaba and Reiss^48^ found that female students felt that they were less able to discuss and perform Physics experiments. Because of that, they felt bored and did not pay attention in class. For this reason, Physics lab environments should be more equitable and inclusive, and support social interactions between students which will reduce gender-biased stereotypes and promote a question of who is Physics for and who can succeed in this field^4,40,49–51^. Therefore, the obtained results indicate that ICBS contributes more than the LIBE approach. It can be assumed that the certainty that the online experiment can be repeated if the circuit is connected incorrectly, without blowing a fuse or damaging the apparatus, i.e. that the error is not clearly visible to the whole class, caused the difference in student motivation.

Also, the results of the second phase of the research showed that the importance of Physics as a science increased more in the ICBS than in the LIBE group. Understanding the importance and place of Physics in education and life causes higher student engagement in class. When student involvement is higher in particular educational conditions, students will invest more time and effort to learn the topic, which will result in their higher performance^7,52^. Also, the ICBS approach caused an increase in the use of active learning strategies, which also supports the change in the attitude of female students toward Physics. Therefore, the significance of the obtained results is seen in the greater inclusion of virtual laboratories in Physics teaching, whose further and mass application can contribute to the change in attitude that Physics is no longer just a “male” subject^53^.

Neither the LIBE nor the ICBS approach caused a statistically significant increase in self-efficacy. The assumption is that there is a deep belief among girls that Physics is not for them. Women and girls in Science are faced with negative stereotypes about them, which negatively impact their belonging to the group^39^. Because Physics identities are directly correlated with self-confidence^6^, it is necessary to apply an inclusive learning environment to prevent discrimination and allow everyone to build up Physics (or science) identities. Woolnough emphases that the teacher’s main focus is to “stimulate the students’ imagination, motivation, and commitment and then to produce self-confidence” in the science subjects^54^. Therefore, the obtained results become more important because they indicate a direction (i.e. the application of the ICBS approach) that, with a longer-term application, could increase female students self-confidence and self-efficacy.

## Conclusions and implications

In this paper, potential gender differences in motivation for learning physics were reviewed, the weakest female motivational components for learning physics were determined, and the effect of different teaching approaches (LIBE and ICBS) on those components and their motivation for learning physics, in general, was determined. The results of the first phase of the research identified differences between male and female students’ motivation to learn Physics. The weakest female motivational components were understanding the importance of Physics as a science and self-efficacy. The results of the second phase show that the approach based on interactive computer simulations (ICBS) is more suitable for females. Therefore, obtained results suggest the larger implementation of the ICBS approach in Physics classrooms as a pedagogical approach that increases female student motivation for learning Physics. The ICBS approach caused less uncertainty in female students’ abilities to conduct the experiment and less fear that male students would laugh at their mistakes, which was more noticeable in the LIBE group. Also, the ICBS approach showed some improvements in understanding the importance of Physics as a science and active learning strategies, which is a big step forward. However, neither the LIBE nor the ICBS approach caused statistically higher self-efficacy. It can be assumed that females’ belief that Physics was not for them was very “deep” and the applied activities were not sufficient to eliminate persistent self-doubts about their abilities. Therefore, further research should focus on longitudinal studies. Also, it seems necessary to include the subjective assessment of girls how they feel during the experiment which they performed in the form of open questionnaires or by monitoring some physiological indicators that can be measured objectively.

## Limitations

Obtained results should be viewed with certain limitations. Classes, where the teacher is female, were observed, therefore the influence of this component was not investigated. Also, the research did not include other external factors, such as parents; their income, education level, marital status, or attitude about girls’ and boys’ education, i.e., exposure to stereotypes. Further research should take into account at least some of these limitations in order to include a greater number of factors and elements for appyling multi-level analyses that can explain the lower representation of girls in science and to find appropriate pedagogical situations that promote greater inclusion of girls in science.

Republic of Serbia, even is traditional patriarchal country, put great effort into this global problem. Therefore, results of this effort is present in Stojanović et al.^55^ which shown that more than 50% teachers from primary and secondary school are female, and number of female students at BS, MSc, and PhD level is the same or higher than man. Therefore, based on positive practice and experiences, the obtained results can be primarily very useful for teachers of all traditional patriarchal countries in order to look the impact of applied teaching approaches on students’ motivation and increase the number of females in science.

## Supplementary Information

Supplementary Information.

## Data Availability

The datasets analyzed during the current study are not publicly available due protection of personal data but are available from the corresponding author on reasonable request.
