# Supplementary material for: Gender motivational gap and contribution of different teaching approaches to female students’ motivation to learn physics
Source: Sci Rep. 2022 Oct 29;12:18224. doi: 10.1038/s41598-022-23151-7 (PMC9617855; doi:10.1038/s41598-022-23151-7)
Supplement: Supplementary file 1 — Supplementary Information. [file 41598_2022_23151_MOESM1_ESM.docx]

**Appendix 1**

Questionnaire for teachers

1. What can you say about female student reactions during the class? How they behaved?

2. Did you talk with them?

3. Did they prepare themselves at home for classes?
